# A Simple Hydrophilic Palladium(II) Complex as a Highly Efficient Catalyst for Room Temperature Aerobic Suzuki Coupling Reactions in Aqueous Media

**DOI:** 10.3390/molecules19056524

**Published:** 2014-05-21

**Authors:** Mengping Guo, Shiwen Liu, Xiuling Zhou, Meiyun Lv, Sanbao Chen, Daoan Xiao

**Affiliations:** 1 Institue of Coordination Catalysis, College of Chemistry and Bio-Engineering, Yichun University, Yichun 336000, China; E-Mails: liushiwen320721@163.com (S.L.); 13879593114@163.com (X.Z.); lvmeiyun2005@163.com (M.L.); 333csb@163.com (S.C.); xiaodaoan2006@163.com (D.X.); 2 Engineering Center of Jiangxi University for Lithium Energy, Yichun University, Yichun 336000, China

**Keywords:** hydrophilic catalyst, aqueous media, green chemistry, Suzuki reaction

## Abstract

A study on room temperature Suzuki cross-coupling in an aqueous medium was carried out using a simple hydrophilic palladium (II) complex, *trans*-PdCl_2_(NH_2_CH_2_COOH)_2_ as catalyst in the presence of K_2_CO_3_ in air. This approach with a comparatively inexpensive and hydrophilic catalyst, mild reaction condition and aqueous media exhibits excellent catalytic activity towards the Suzuki coupling of aryl bromides and arylboronic acids, and good yields were obtained in the Suzuki coupling of activated aryl chlorides.

## 1. Introduction

During the last two decades, the palladium-catalyzed Suzuki-Miyaura cross-coupling reaction between aryl halides and arylboronic acids is probably one of the most studied by the chemists. Its discovery and industrial significance were recognized by the 2010 Nobel Prize [1,2,3,4]. From the academic and industrial view points, alternative reaction media are currently of considerable interest given an increasing emphasis on making this palladium catalyzed cross-coupling process “greener” [5,6,7], for example, by minimizing the use of organic solvents [8,9]. Water is the obvious leading candidate in this regard because of its low cost, non-flammability, non-toxicity and low environmental concerns [10,11,12]. Since the first use of water in palladium-catalyzed Suzuki cross-coupling reactions was reported by Casalnuovo and Calabrese in 1990, a large number of strategies for Suzuki-Miyaura cross-coupling reactions in water have been developed [13,14,15,16,17,18], such as synthesizing water-soluble catalysts or water-soluble ligands [19,20,21,22,23,24,25], adding surfactants or phase-transfer agents [26,27,28,29,30], using organic co-solvents or inorganic salts as a promoter [31,32], utilizing microwave-heating or ultrasonic-irradiation [33,34,35,36]. During our ongoing research on the Suzuki coupling reaction [37,38,39,40,41], we have been interested in the design of new, highly efficient, air- and moisture-stable palladium catalysts that can be used in room temperature Suzuki cross-coupling reaction in organic/aqueous co-solvents medium under ambient atmosphere since such catalysts have the potential to be used in industrial systems. It is also desirable to have simple, inexpensive, easily accessible and stable catalysts for this reaction. In 2003, Boykin *et al.*, for the first time, report the first well-defined single-component Pd(OAc)_2_-amine complex as an efficient catalyst and its temperature-dependent catalytic behavior toward aryl bromides with different electronic substituents [42]. Herein, we report an air- and moisture-stable hydrophilic palladium(II) complex **2** (Scheme 1) containing the simple, economical and accessible glycine ligand **1** for palladium-catalyzed fast room temperature Suzuki coupling reactions of arylboronic acids with aryl halides in aqueous media under an ambient atmosphere.

## 2. Results and Discussion

The palladium(II) complex **2**, which is water-soluble and air-stable, was synthesized according to the reaction illustrated in Equation (1):

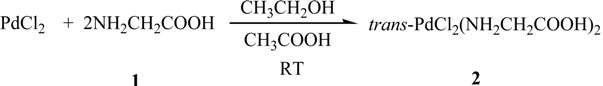
(1)


The next investigation was to study the catalytic activity of the synthesized complex **2** in Suzuki cross-coupling reactions. With the water-soluble catalyst PdCl_2_(NH_2_CH_2_COOH)_2_ at hand, we performed the Suzuki cross-coupling between bromobenzene and phenylboronic acid in the presence of 1.0 mol % of **2** in water without organic co-solvent at room temperature in 20 h for screening different bases. The results are summarized in Table 1. Unfortunately, inorganic bases, which are soluble in water, demonstrated low activity in the present protocol (Table 1, entries **1**–**7**). It is a well-known fact that many reports have described use of alcohol as co-solvent in the Suzuki-Miyaura reaction [43,44,45,46,47,48]. Therefore we studied the use of alcohol as co-solvent in this protocol. As expected, we were delighted to see that the ethanol/water (in 1:1 proportion) used had a dramatic effect on the reactivity of K_2_CO_3_ and the desired biphenyls were isolated in nearly quantitative yields within 30 min with complex **2** as catalyst at room temperature under aerobic conditions (Table 1, entries **9**). Among the alcohols screened, propanol and isopropanol gave good turnovers (Table 1, entries **10**–**11**). The lowest yields (24%, 18%) were obtained in methanol, and *n*-butyl alcohol (Table 1, entries **8**, **12**). Consequently, ethanol/water (in 1:1 proportion) was chosen as the best solvent.

**Table 1:** The effects of base and solvent on the cross-coupling of bromobenzene and phenylboronic acid. ^a^ 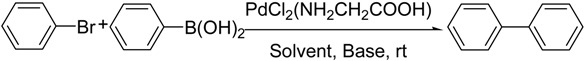

Entry	Solvent	Base	Yield ^b^ (%)
1	Water	Na_2_CO_3_	39
2	Water	K_2_CO_3_	49
3	Water	Na_3_PO_4_	26
4	Water	NaOAc	30
5	Water	Cs_2_CO_3_	35
6	Water	NaF	43
7	Water	NaOH	40
8 ^c^	Methanol + Water	K_2_CO_3_	24
9 ^c^	Ethanol + Water	K_2_CO_3_	99
10 ^c^	Propanol + Water	K_2_CO_3_	76
11 ^c^	Isopropanol + Water	K_2_CO_3_	85
12 ^c^	N-Butyl alcohol + Water	K_2_CO_3_	18

^a^ Reaction conditions: carried out with 1 mmol of bromobenzene, 1.2 mmol of phenylboronic acid and 0.01 mmol of PdCl_2_(NH_2_CH_2_COOH)_2_ in 4 mL of H_2_O for 20 h, room temperature; ^b^ Isolated yield was based on the bromobenzene; ^c^ Carried out with 1 mmol of bromobenzene, 1.2 mmol of phenylboronic acid and 0.01 mmol of PdCl_2_(NH_2_CH_2_COOH)_2_ in 6 mL of alcohol/H_2_O (in 1:1 proportion) for 30 min, room temperature.

We next evaluated the scope and limitations of the current procedure for Suzuki-Miyaura reactions between aryl halides and arylboronic acids in the presence of 1 mol % PdCl_2_(NH_2_CH_2_COOH)_2_ and two equivalents of K_2_CO_3_ at room temperature in ethanol/water (in 1:1 proportion). The results are shown in Table 2. Aryl bromides with various functional groups efficiently reacted with phenylboronic acid affording the desired biphenyls in good to excellent conversions (Table 2, entries **1**–**3**). In the case of *o*-bromotoluene with a sterically encumbering substituent the conversion is good (Table 2, entries **5**, **8**), but 2-bromo*-m*-xylene with a di-*ortho*-substituent react reluctantly with phenylboronic acid and a very poor yield was obtained (Table 2, entry **9**). The effect of varying the arylboronic acids was studied under similar experimental condition. The arylboronic acids with electron-donating groups afforded the corresponding coupling products in good to excellent yields (Table 2, entries **4**, **6**, **7**), while slightly lower conversions were observed when arylboronic acids containing electron-withdrawing substituents are used (Table 2, entries **10**, **11**).

In general aryl chlorides are difficult substrates for coupling reactions because of the stronger C-Cl bond strength, so next, we turned our attention to the Suzuki-Miyaura reactions of aryl chlorides. Significantly catalyst **2** was able to coupling electron-withdrawing aryl chlorides with arylboronic acids and gave the desired products in moderate yields within 4 h (Table 2, entries **12**–**15**). It is worthy of mention that although relatively less yields of coupling products with aryl chlorides have been obtained compared to aryl bromides as substrates, these results are still quite significant, as they represent successful examples of the Suzuki-Miyaura reaction of activated aryl chlorides at room temperature using environmentally-benign reaction media. Unfortunately, this reaction proceeded sluggishly for electron-neutral and electron-releasing aryl chlorides as substrates, such as *para*-chlorotoluene, giving biaryls in only 15% yield.

**Table 2:** The Suzuki-Miyaura reactions of aryl halides with arylboronic acids. ^a^ 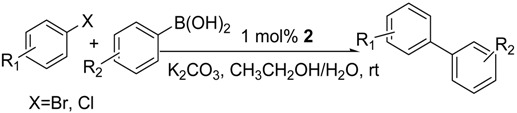

Entry	Aryl halide		Arylboronic acid		Product		Yield ^b^ (%)
**1**	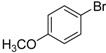		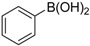		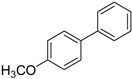		94
**2**	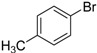		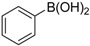		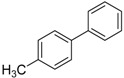		90
**3**	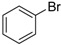		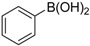		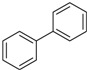		99
**4**	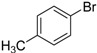		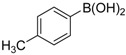		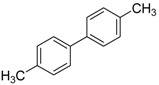		95
**5**	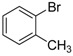		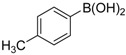		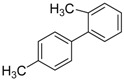		86
**6**	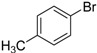		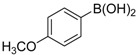		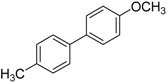		94
**7**	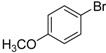		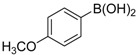		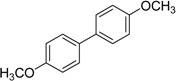		99
**8**	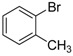		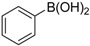		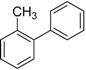		88
**9**	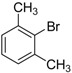		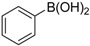		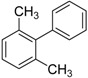		32
**10**	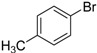		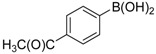		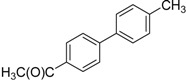		68
**11**	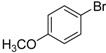		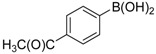		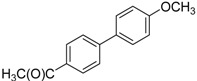		64
**12** ^c^	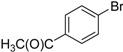		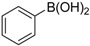		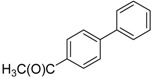		77
**13** ^c^	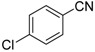		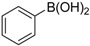		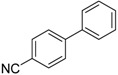		73
**14** ^c^	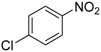		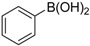		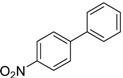		69
**15** ^c^	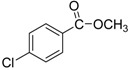		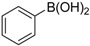		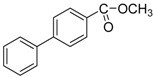		66

^a^ Carried out with 1 mmol of aryl bromide, 1.2 mmol of arylboronic acid and 0.01 mmol of PdCl_2_(NH_2_CH_2_COOH)_2_ in 6 mL of CH_3_CH_2_OH/H_2_O (in 1:1 proportion) for 30 min, room temperature, in air; ^b^ Isolated yield was based on the aryl halide; ^c^ Carried out with 1 mmol of aryl chloride, 1.2 mmol of arylboronic acid and 0.01 mmol of PdCl_2_(NH_2_CH_2_COOH)_2_ in 6 mL of CH_3_CH_2_OH/H_2_O (in 1:1 proportion) for 4 h, room temperature, in air.

## 3. Experimental

### 3.1. General Information

All chemicals employed in the synthesis were analytical grade, obtained commercially from Aldrich (St Louis, MO, USA) or Alfa Aesar (Ward Hill, MA, USA) and used as received without any prior purification. Silica gel 60 GF254 was used for analytical TLC. ^1^H-NMR, ^13^C-NMR spectra were recorded on a Bruker Avance III (400 MHz) spectrometer using tetramethylsilane as the internal standard and CDCl_3_ or DMSO as the solvent. Elemental analyses (C, H, N) were carried out on a Perkin Elmer model 240 C automatic instrument. Electrospray mass spectra (ES-MS) were recorded on a Finnigan LCQ mass spectrometer.

### 3.2. General Procedure for the Preparation of the Hydrophilic Palladium(II) Complex **2**

Glycine (**1**, 10.2 mmol) was dissolved in glacial acetic acid (20 mL) and a concentrated ethanolic solution of the palladium chloride (5.0 mmol) was added. After several hours the crystalline product **2** was collected, washed with acetic acid and with ether and dried at 250/1 mm Hg to obtain a light brown solid. Yield: 0.93 g, 57%. Calcd. for C_4_H_10_N_2_Cl_2_O_4_Pd(%): C 14.67, H 3.08, N8.55; found: C 14.90, H 3.10, N 8.56; ESI-MS (*m/z*): 326.8 [ M+H ]^+^. The geometry of this hydrophilic palladium(II) complex PdCl_2_(NH_2_CH_2_COOH)_2_ is *trans*, according to Jarolìm’s report [49].

### 3.3. General Procedure for the Suzuki Type Coupling Reactions

All Suzuki reactions were carried out without an inert atmosphere. A mixture of aryl halide (1.0 mmol), arylboronic acid (1.2 mmol), K_2_CO_3_ (2 mmol), and PdCl_2_(NH_2_CH_2_COOH)_2_ (0.01 mmol) in CH_3_CH_2_OH/H_2_O (in 1:1 proportion, 6 mL) was allowed to react at room temperature for 30 min, and then quenched with brine (15 mL) and extracted three times with ethyl acetate (3 × 15 mL). The combined organic phase was dried with MgSO_4_, filtrate, solvent was removed on a rotary evaporator, and the product was isolated by thin layer chromatography. The purified products were identified by ^1^H-NMR and ^13^C-NMR spectroscopy.

### 3.4. Analytical Data of Representative Products

*4,4'-Dimethylbiphenyl* (Table 2, entry **4**): ^1^H-NMR (CDCl_3_) δ 7.54 (d, *J* = 8.0 Hz, 4H), 7.29 (d, *J* = 7.2 Hz, 4H), 2.45 (s, 6H). ^13^C-NMR: δ 21.1, 126.8, 129.5, 136.7, 138.3.

*4-Acetyl-4'-methylbiphenyl* (Table 2, entry **10**): ^1^H-NMR (CDCl_3_) δ 8.05 (d, *J* = 8.0 Hz, 2H), 7.70 (d, *J* = 8.0 Hz, 2H), 7.56 (d, *J* = 8.4 Hz, 2H), 7.31 (d, *J* = 8.0 Hz, 2H), 2.66 (s, 3H), 2.44 (s, 3H). ^13^C-NMR: δ 21.2, 26.7, 127.0, 127.1, 128.9, 135.6, 137.0, 138.3, 145.7, 197.8.

*4-Acetyl-4'-methoxybiphenyl* (Table 2, entry **11**): ^1^H-NMR (CDCl_3_) δ 8.00 (d, *J* = 8.0 Hz, 2H), 7.64 (d, *J* = 8.4 Hz, 2H), 7.58 (d, *J* = 8.8 Hz, 2H), 7.00 (d, *J* = 8.8 Hz, 2H), 3.86 (s, 3H), 2.63 (s, 3H). ^13^C-NMR: δ 26.6, 55.4, 114.4, 126.6, 128.4, 129.0, 132.3, 135.3, 145.4, 160.0, 197.7.

## 4. Conclusions

In conclusion, a convenient and fast methodology has been developed for the palladium-catalyzed aerobic Suzuki-Miyaura reaction in aqueous media at room temperature using a simple, inexpensive, easily accessible, stable and hydrophilic palladium (II) complex, PdCl_2_(NH_2_CH_2_COOH)_2_ as catalyst. The advantages of our catalytic system compared with Boykin’s reported one [42] rest on the application of unactivated aryl bromides and activated aryl chlorides at room temperature. This method is consistent with the green chemistry concept and provides both an economically and technologically practical procedure for the synthesis of complexes containing the biaryl nucleus in industrial applications.

## Supplementary Materials

Supplementary materials can be accessed at: http://www.mdpi.com/1420-3049/19/5/6524/s1.
