# Supplementary material for: A Simple Hydrophilic Palladium(II) Complex as a Highly Efficient Catalyst for Room Temperature Aerobic Suzuki Coupling Reactions in Aqueous Media
Source: Molecules. 2014 May 21;19(5):6524–33. doi: 10.3390/molecules19056524 (PMC6270763; doi:10.3390/molecules19056524)

Supplementary Materials

1. Characterization of Palladium(II) Complex 2

Figure S1. ES-MS spectrum of Palladium(II) Complex **2**.

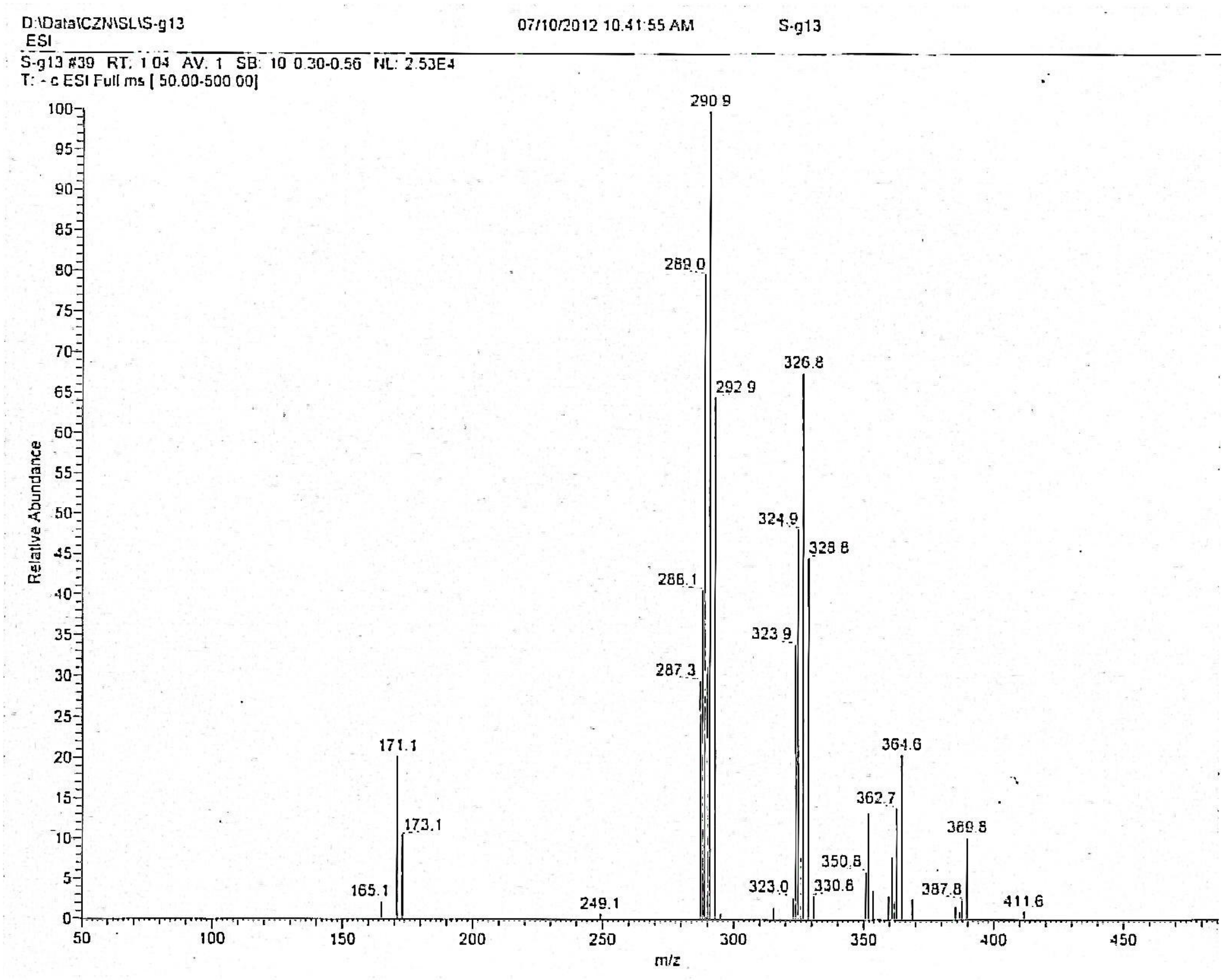

2. Characterization of the Products

Figure S2. Copy of ^{1}H-NMR for 4,4'-dimethylbiphenyl.

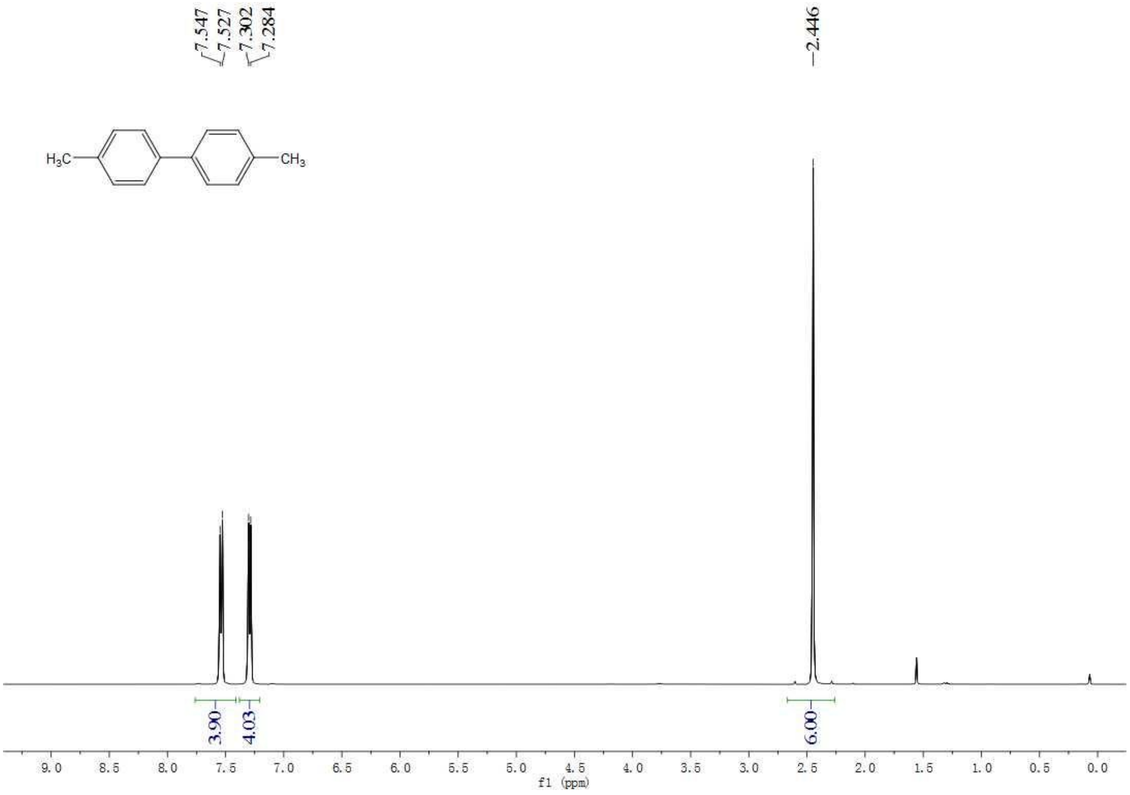

Figure S3. Copy of ^{13}C-NMR for 4,4'-dimethylbiphenyl.

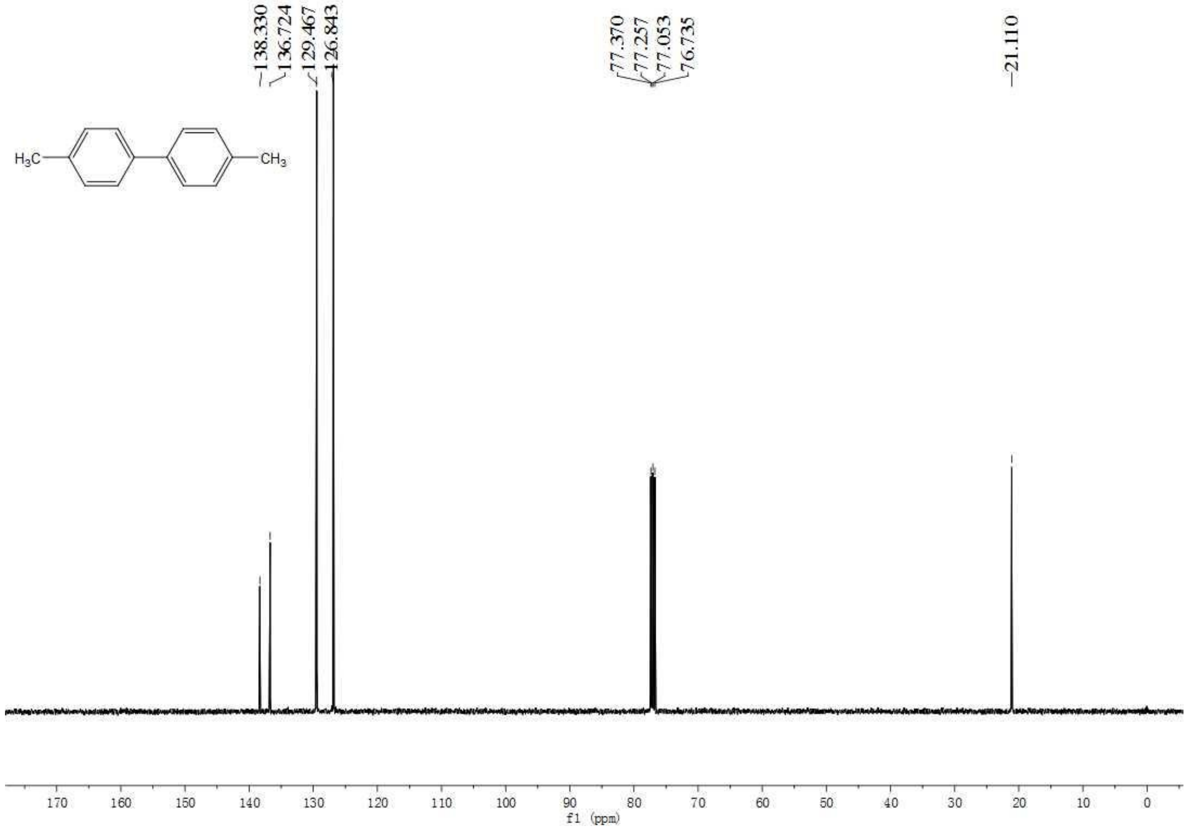

Figure S4. Copy of ^{1}H-NMR for biphenyl.

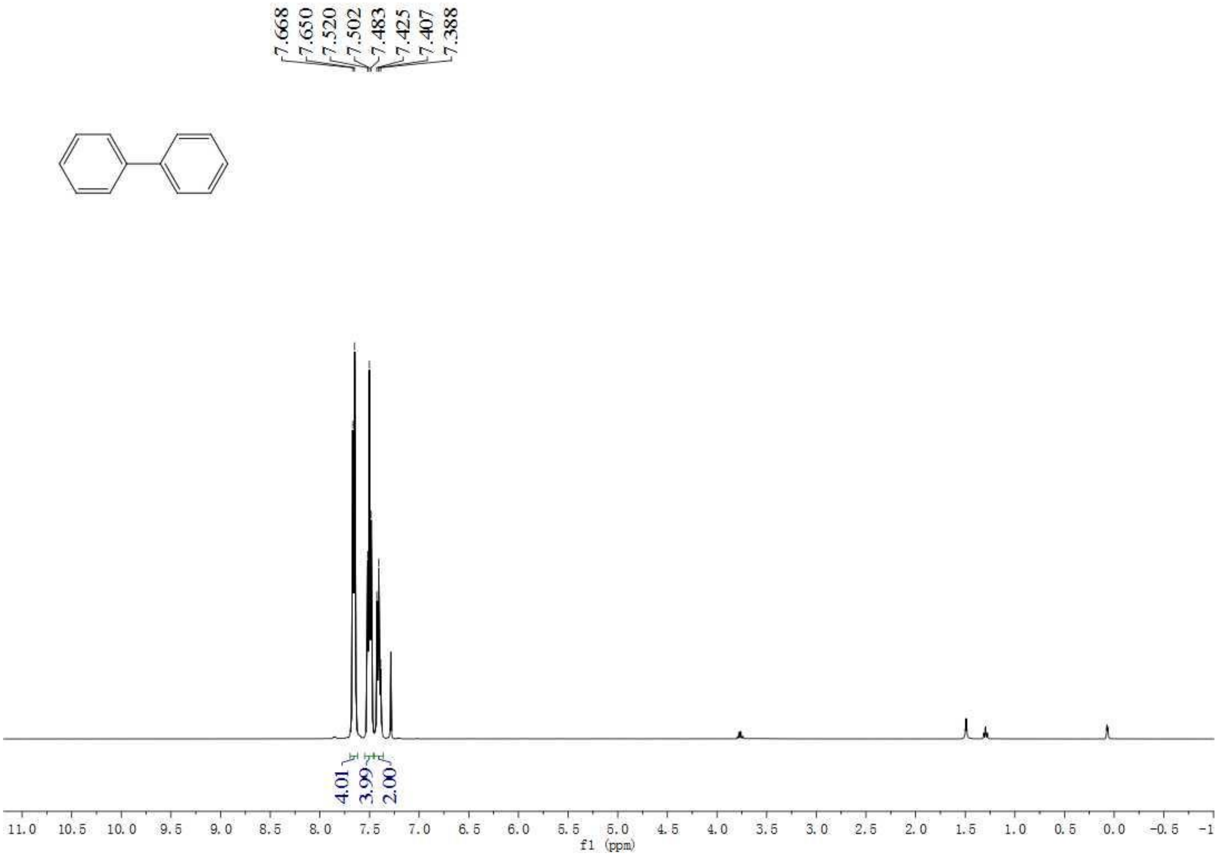

Figure S5. Copy of ^{13}C-NMR for biphenyl.

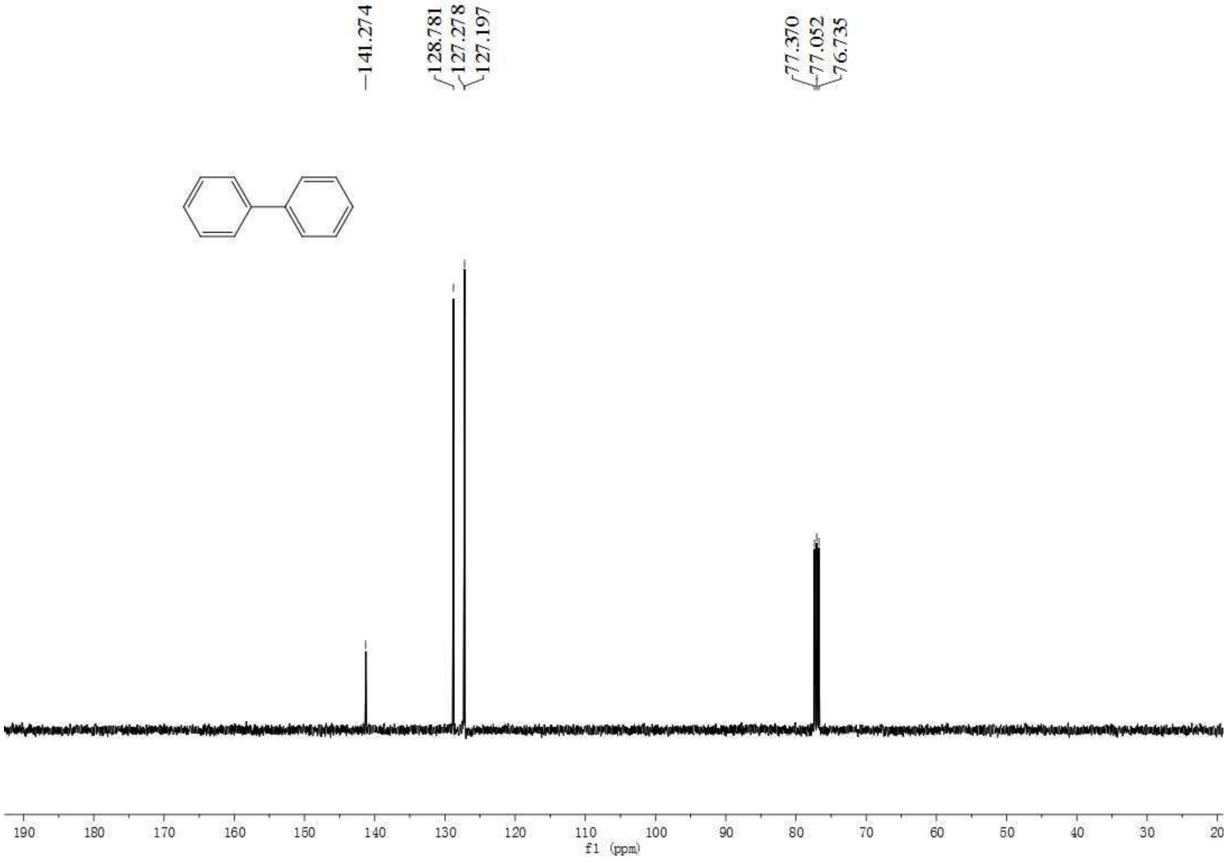

Figure S6. Copy of ^{1}H-NMR for 4-methoxybiphenyl.

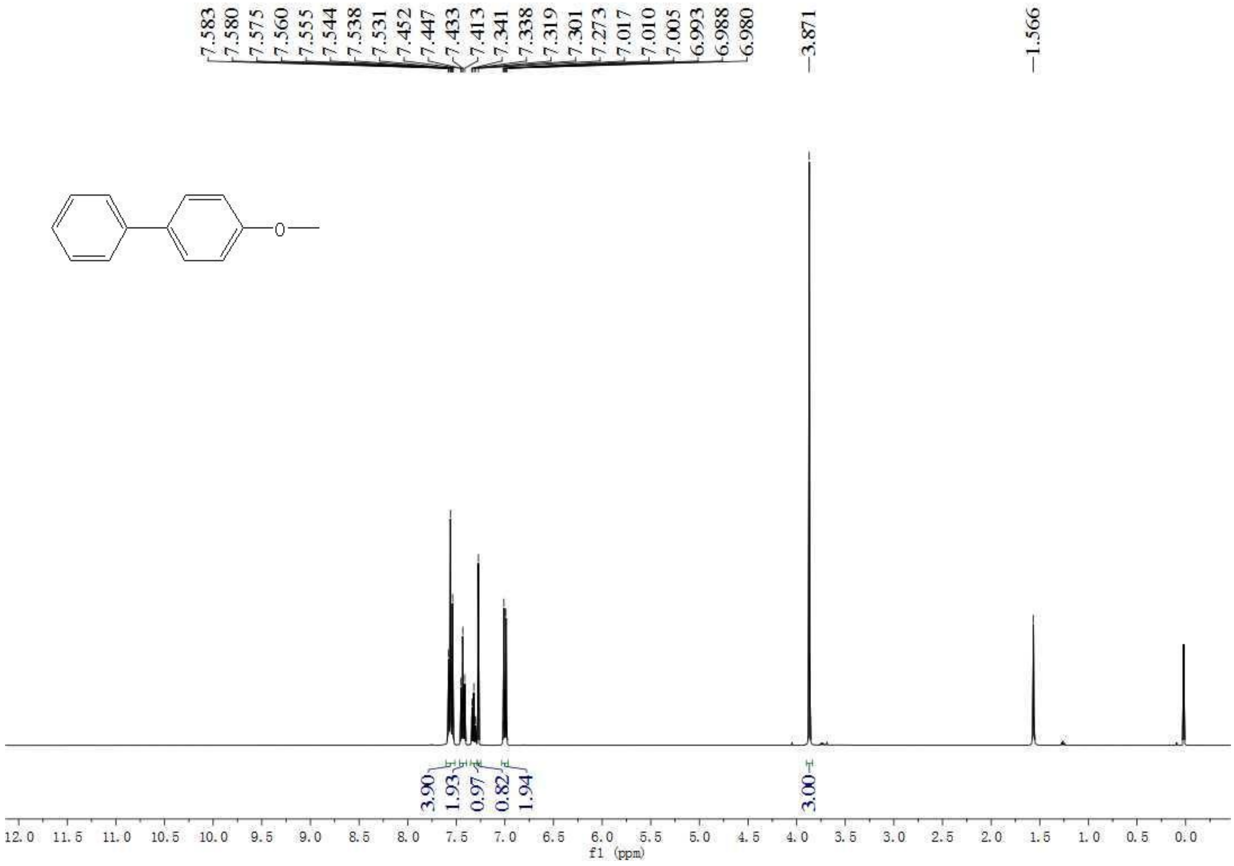

Figure S7. Copy of ^{13}C-NMR for 4-methoxybiphenyl.

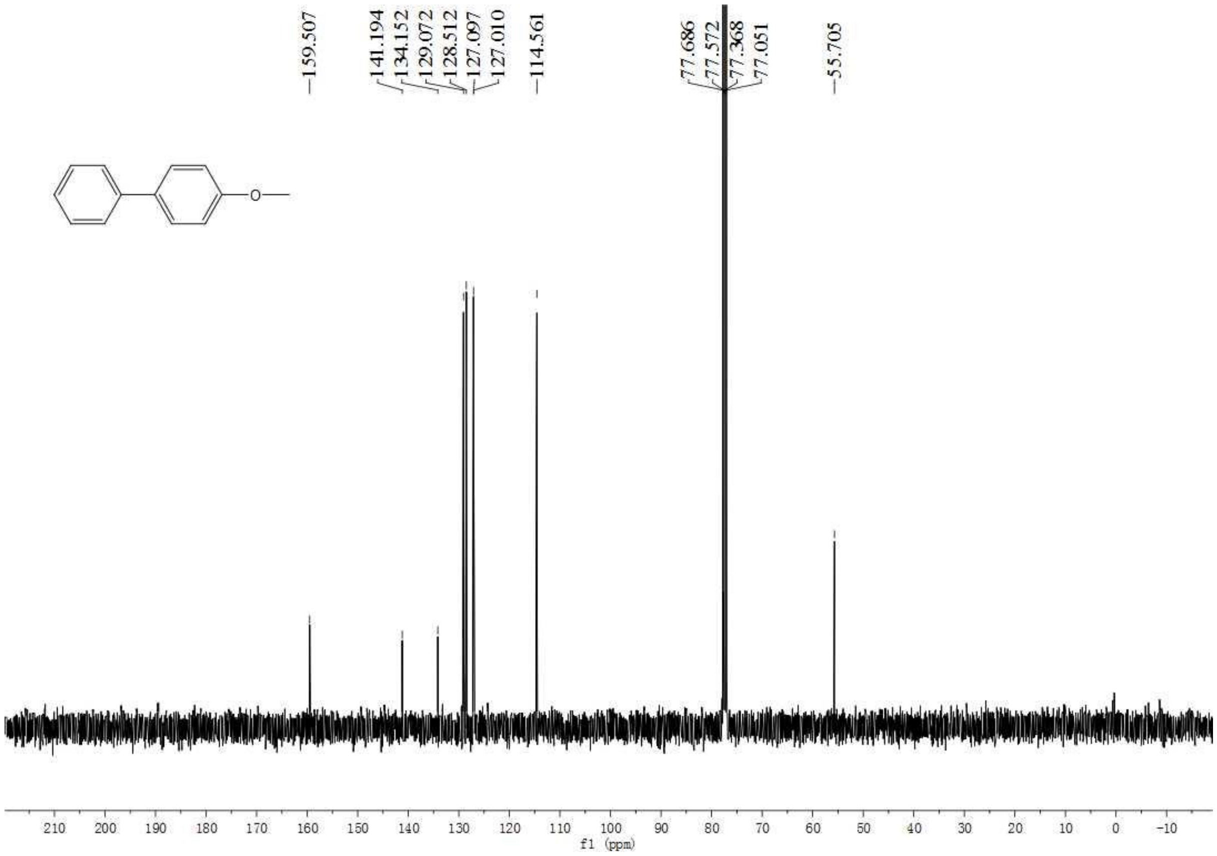

Figure S8. Copy of ^{1}H-NMR for 4-methylbiphenyl.

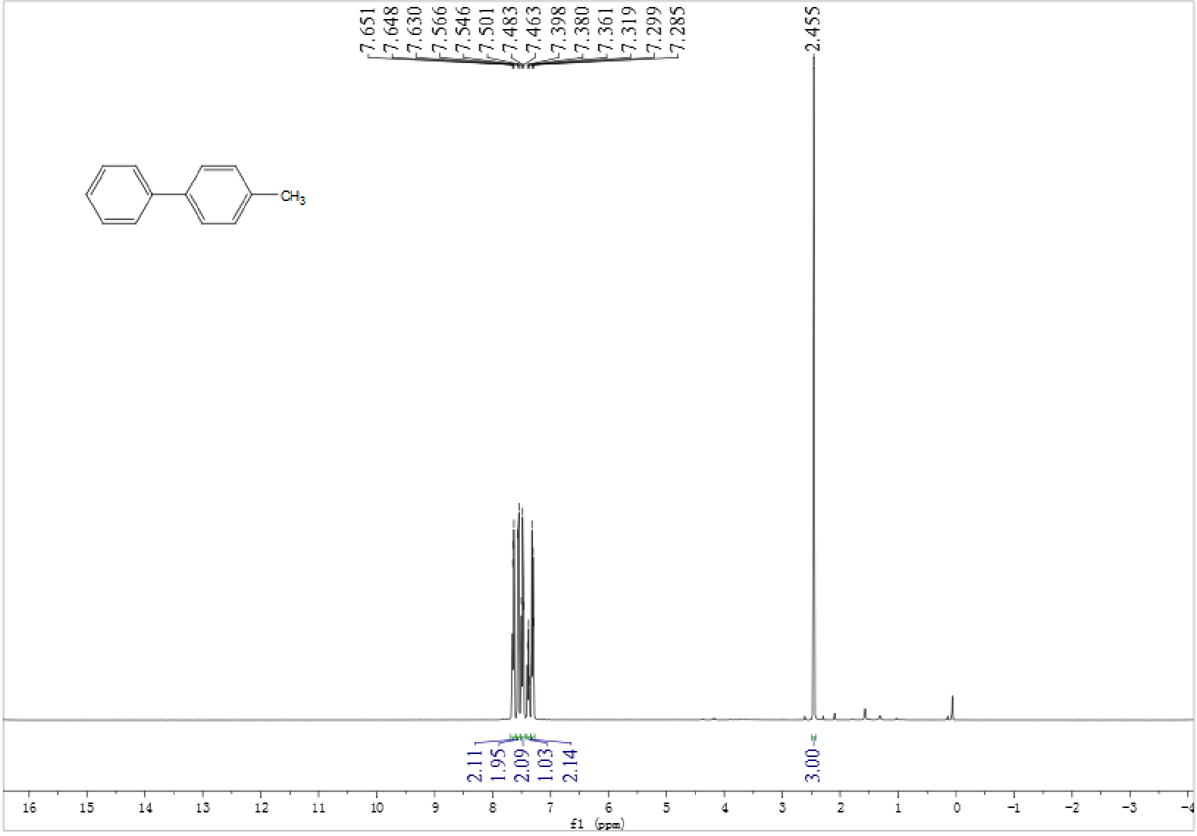

Figure S9. Copy of ^{13}C-NMR for 4-methylbiphenyl.

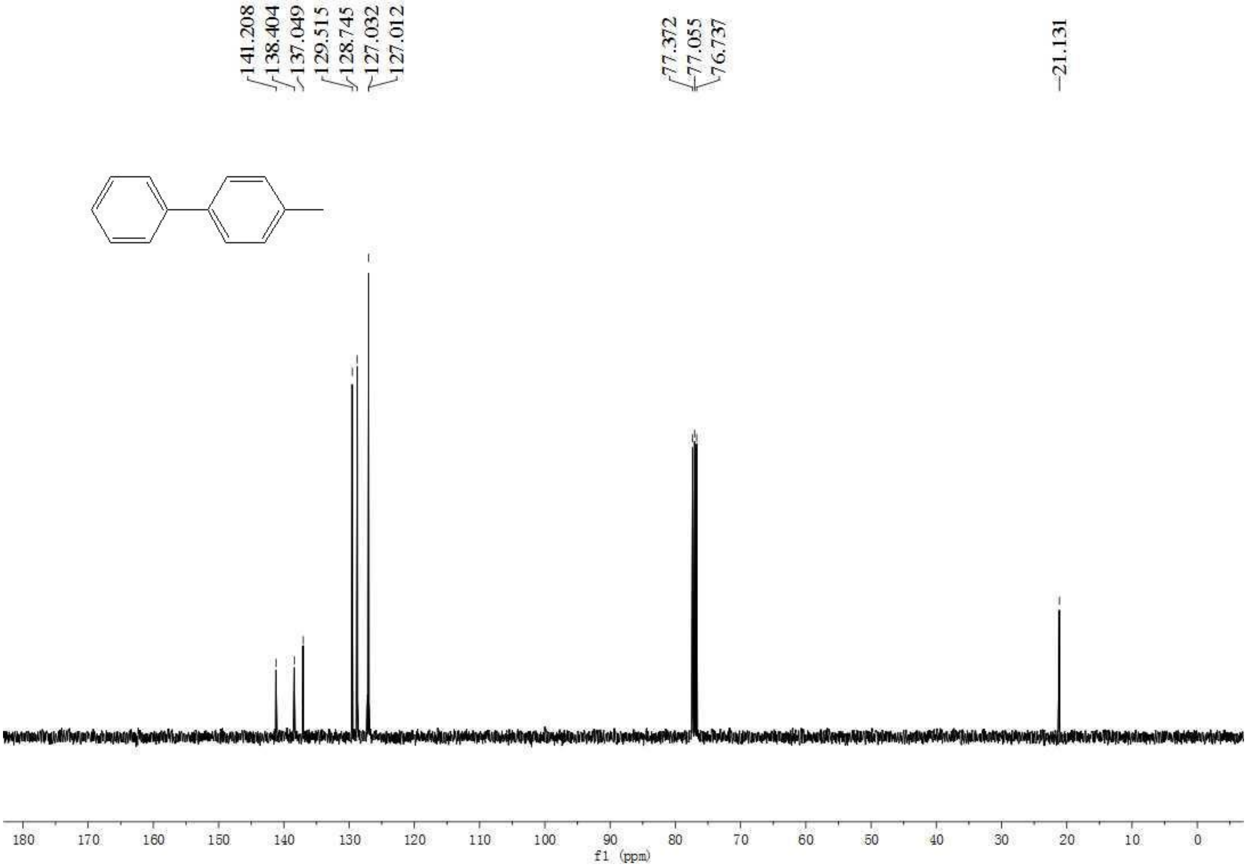

Figure S10. Copy of ^{1}H-NMR for 4-acetyl-4'-methylbiphenyl.

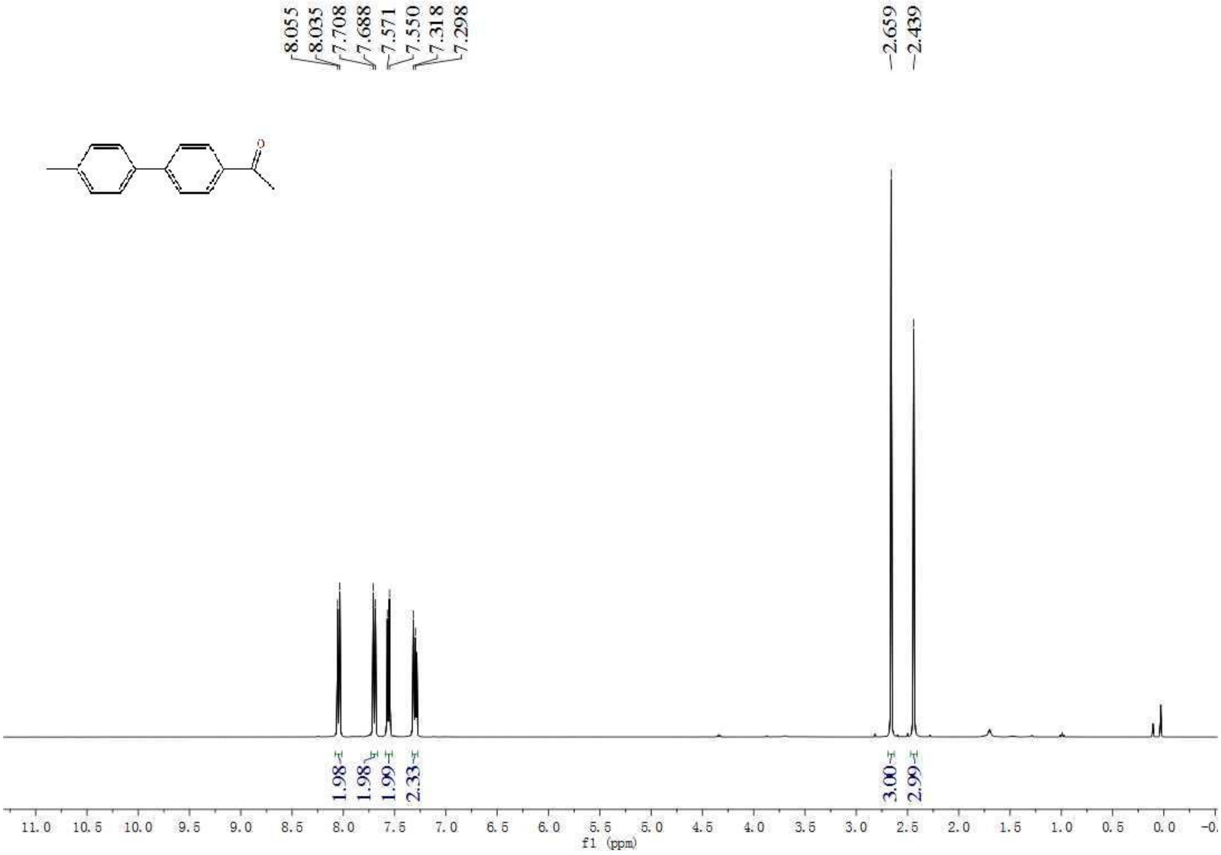

Figure S11. Copy of ^{13}C-NMR for 4-acetyl-4'-methylbiphenyl.

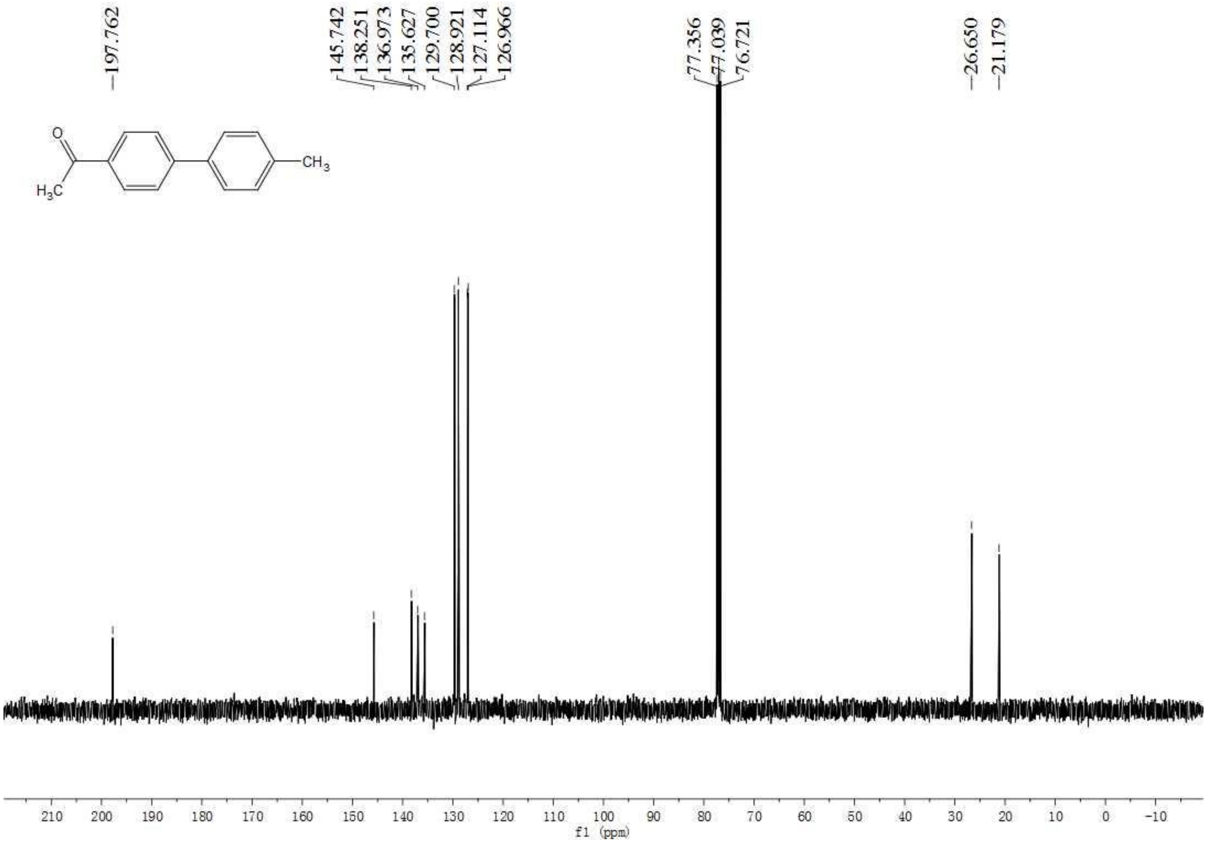

Figure S12. Copy of ^{1}H-NMR for 4-acetyl-4'-methoxybiphenyl.

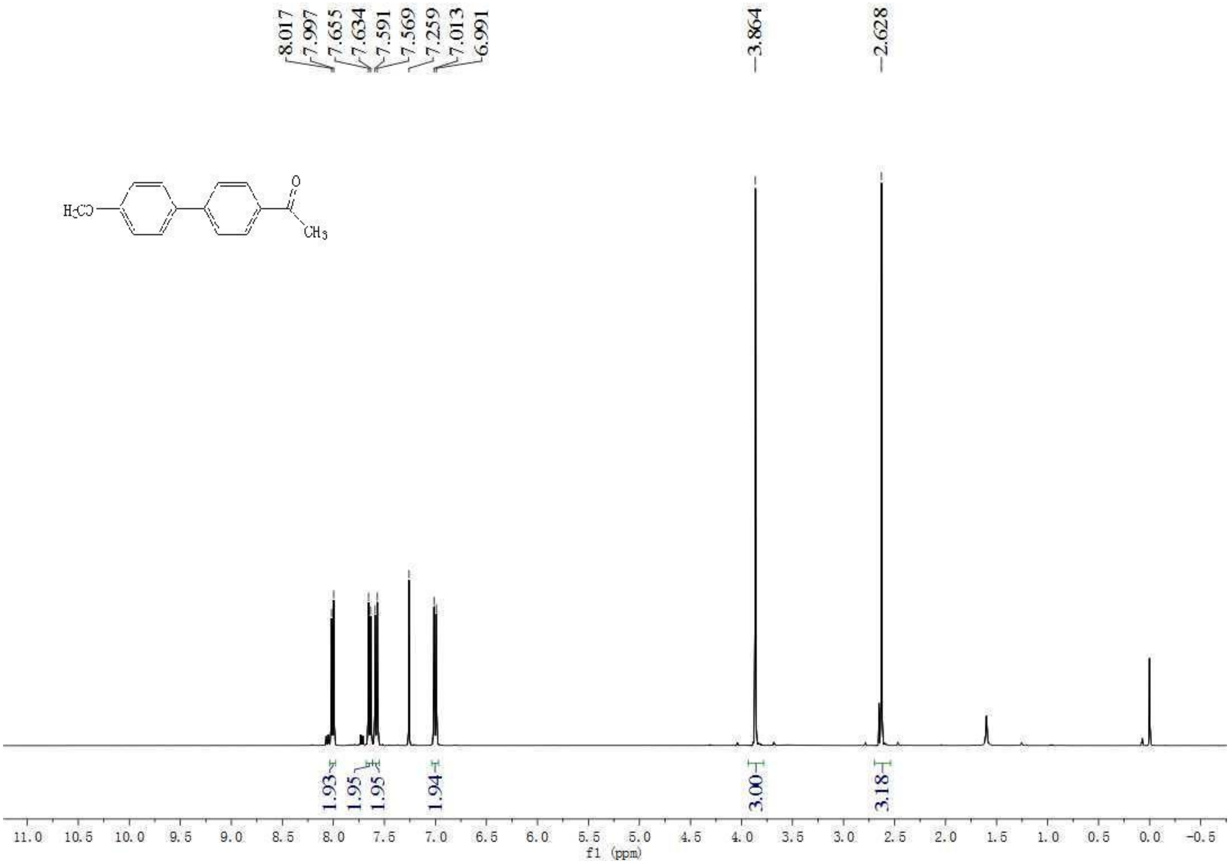

Figure S13. Copy of ^{13}C-NMR for 4-acetyl-4'-methoxybiphenyl.

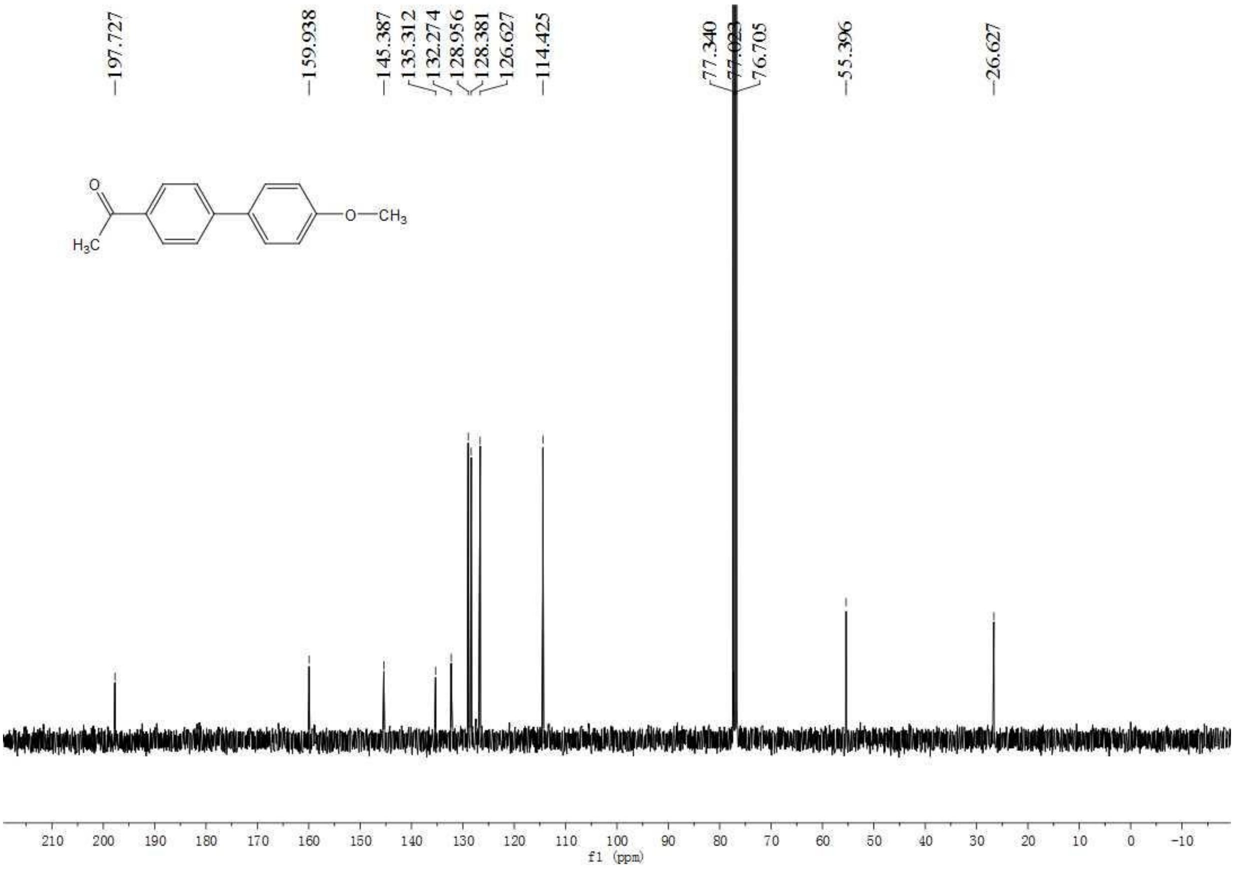

Figure S14. Copy of ^{1}H-NMR for 2-methylbiphenyl.

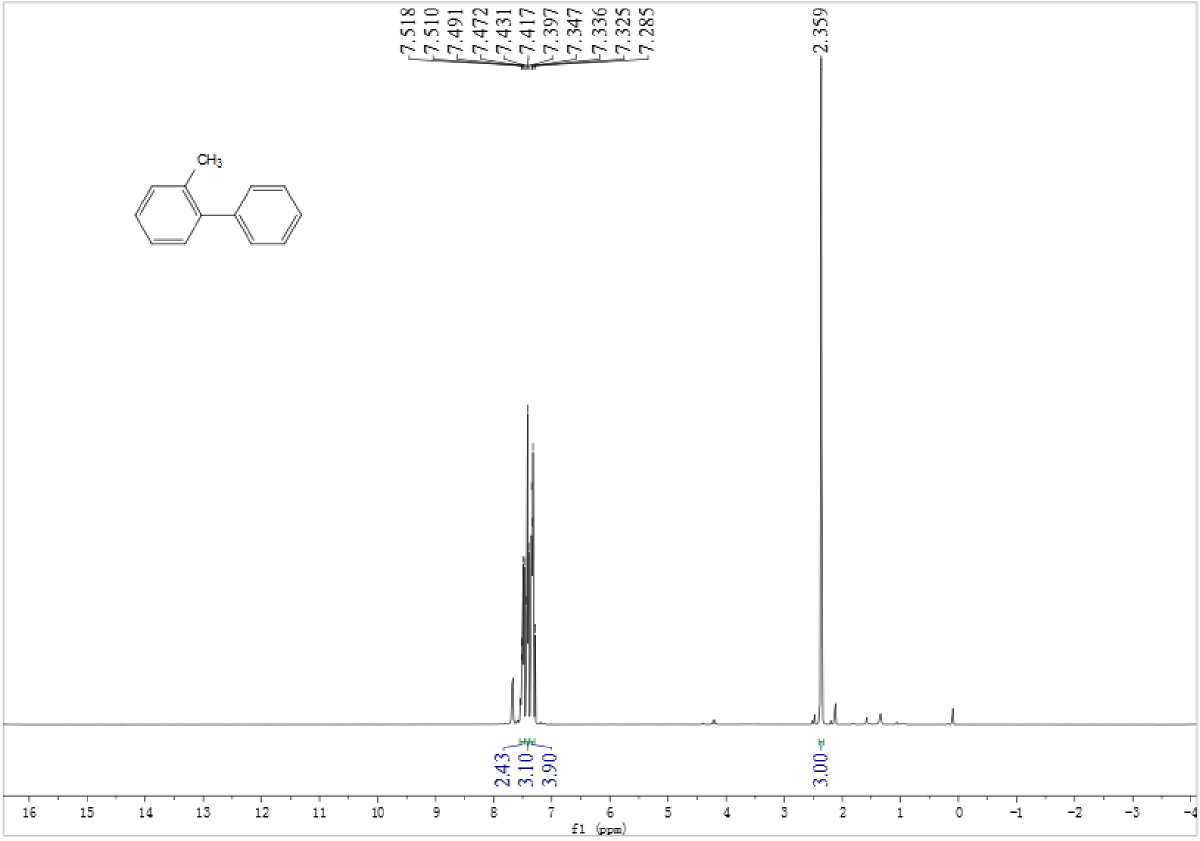

Figure S15. Copy of ^{13}C-NMR for 2-methylbiphenyl.

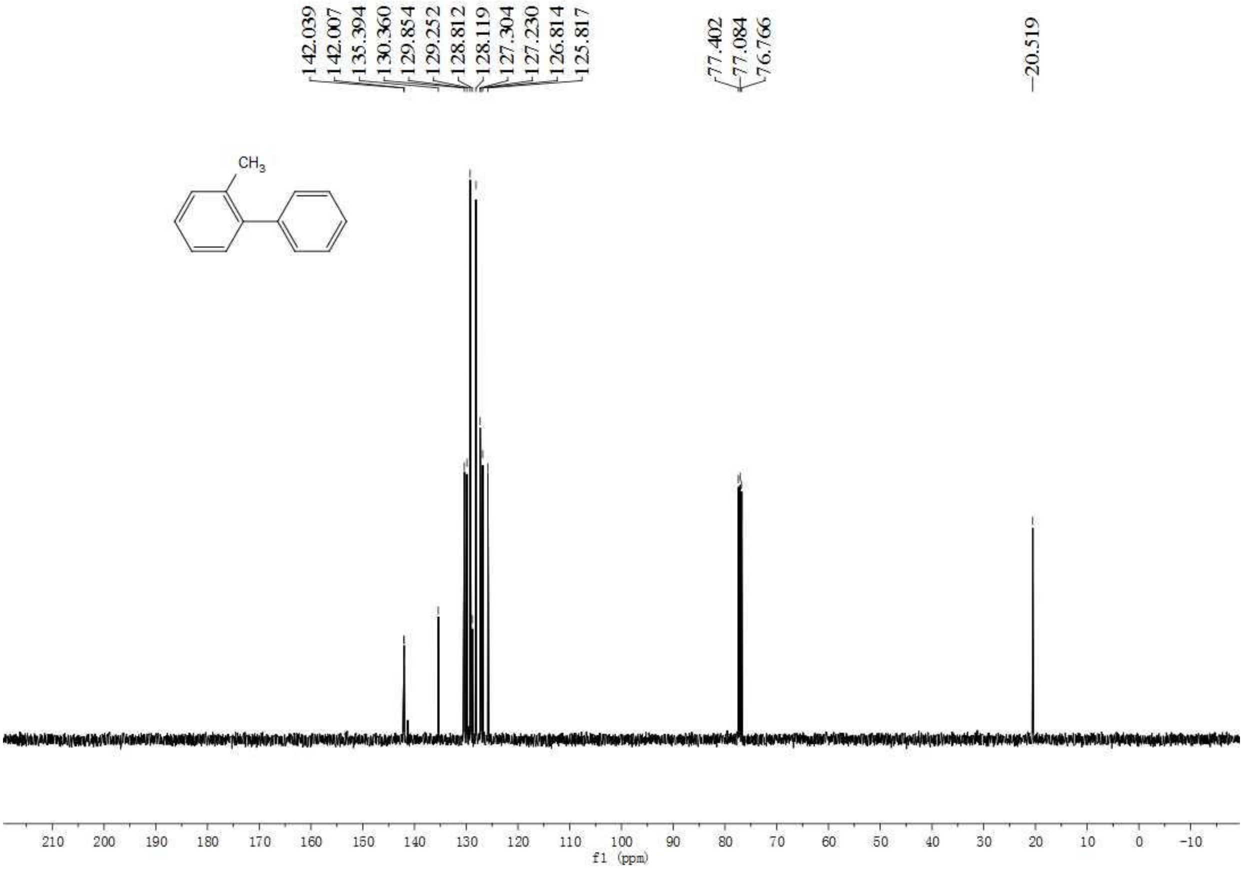

Figure S16. Copy of ^{1}H-NMR for 2,4'-dimethylbiphenyl.

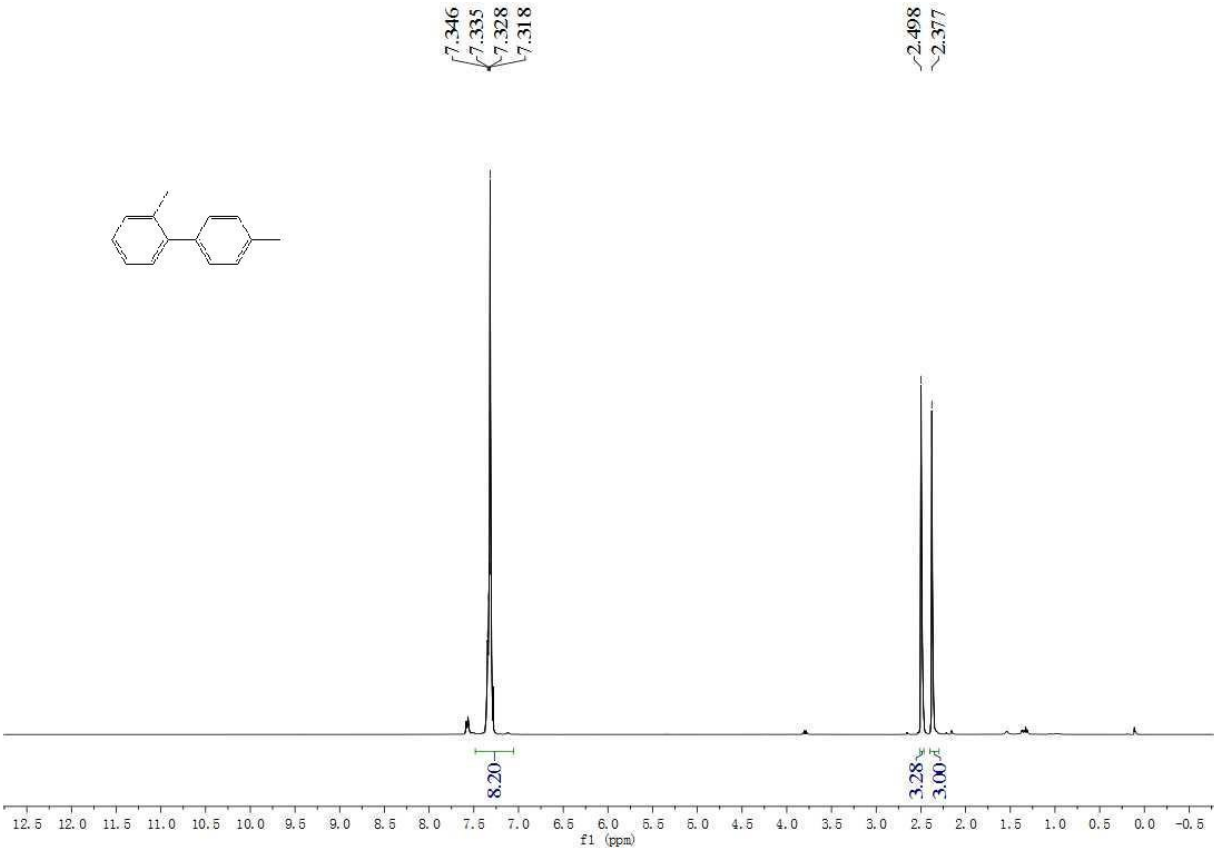

Figure S17. Copy of ^{13}C-NMR for 2,4'-dimethylbiphenyl.

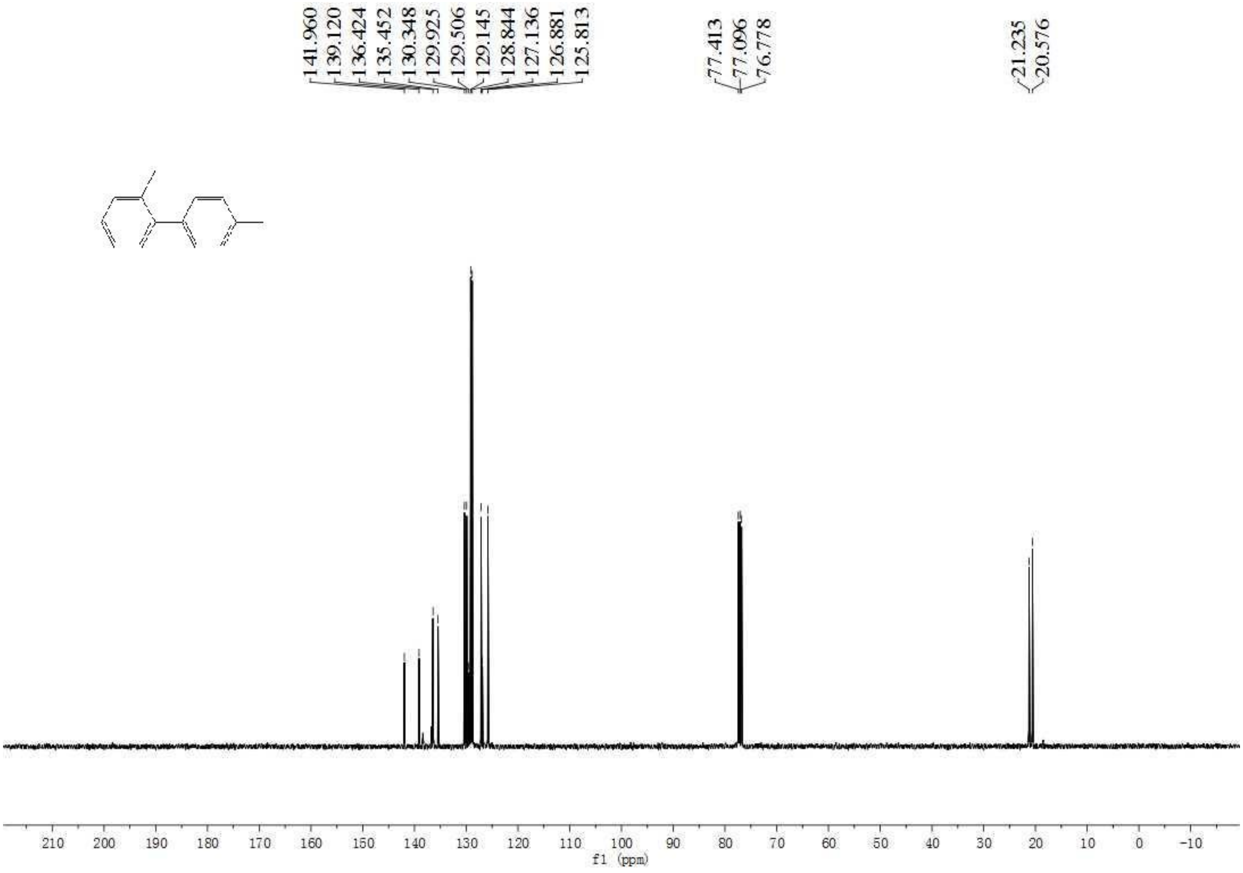

Figure S18. Copy of ^{1}H-NMR for 4,4'-dimethoxybiphenyl.

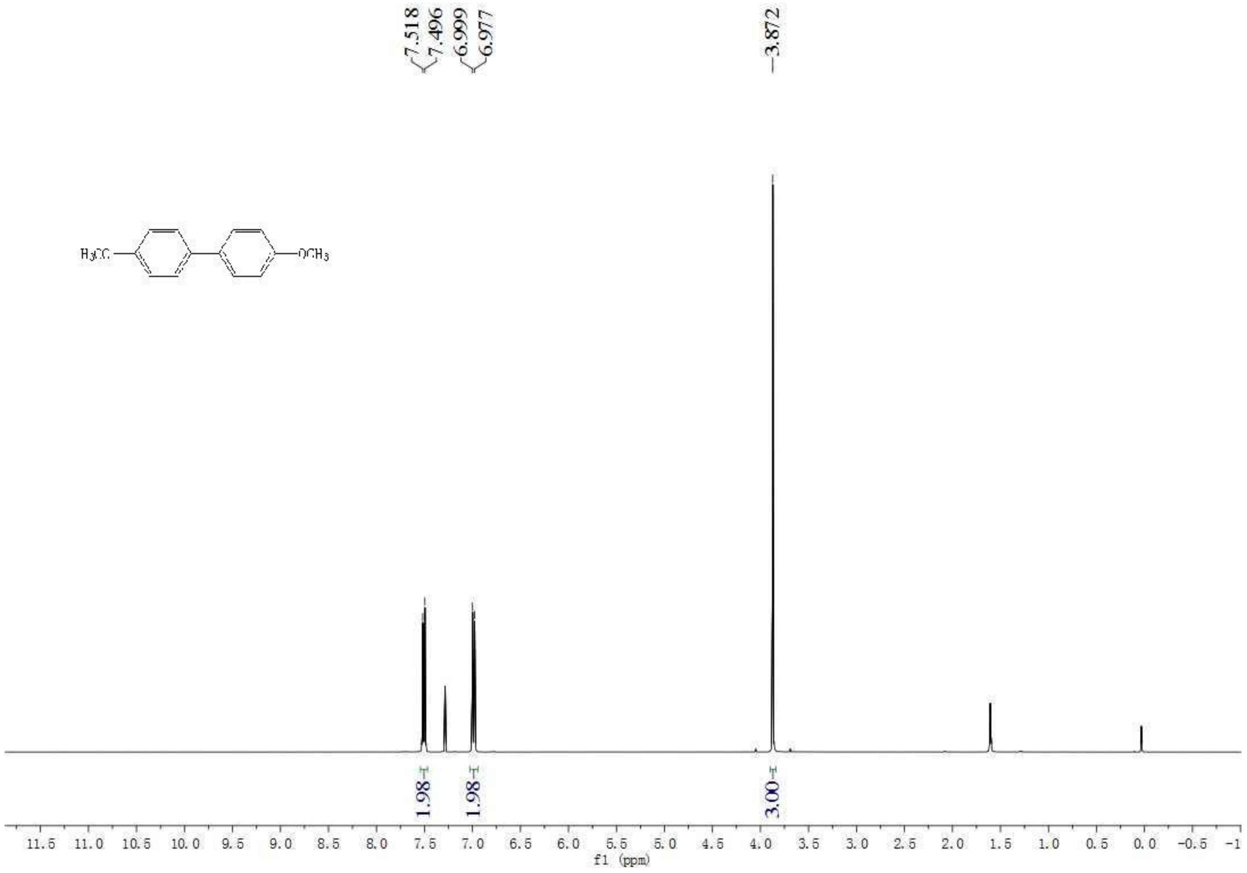

Figure S19. Copy of ^{13}C-NMR for 4,4'-dimethoxybiphenyl.

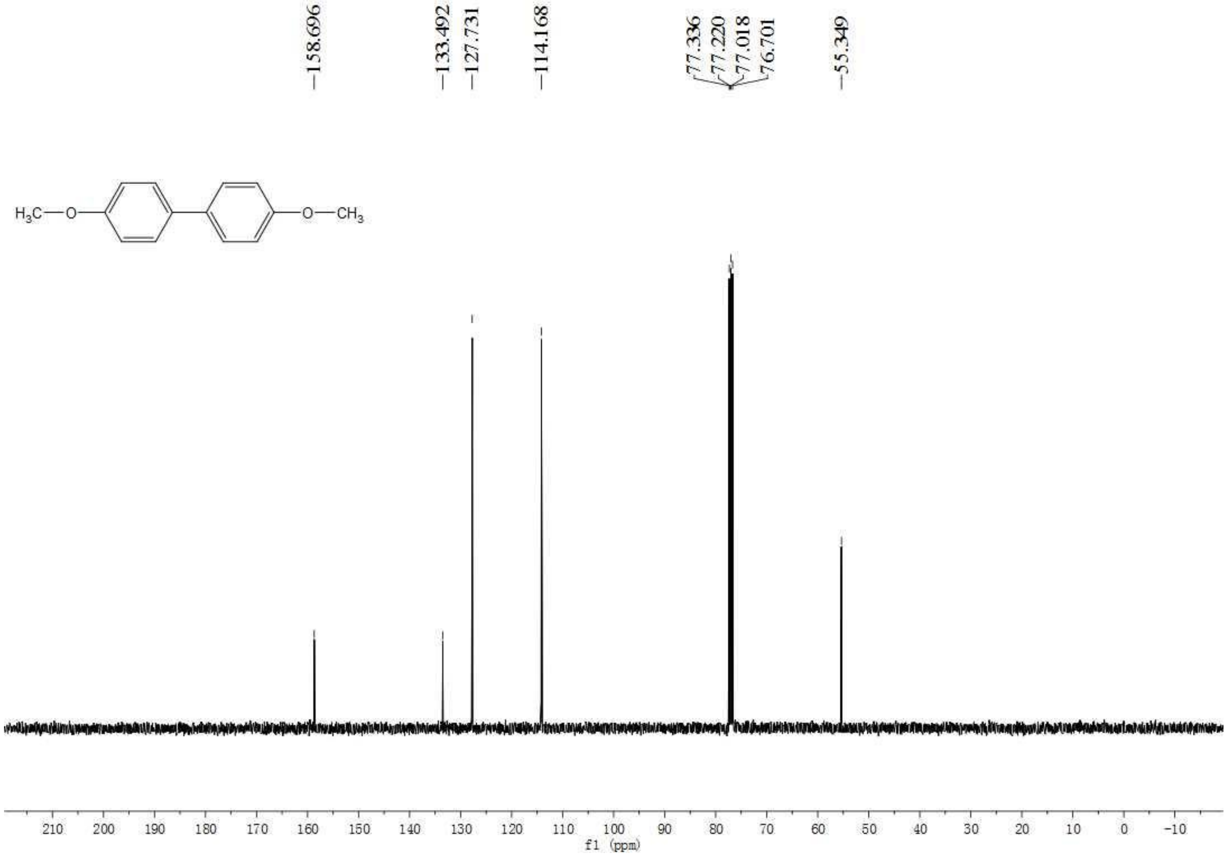

Figure S20. Copy of ¹H-NMR for 4-cyanobiphenyl.

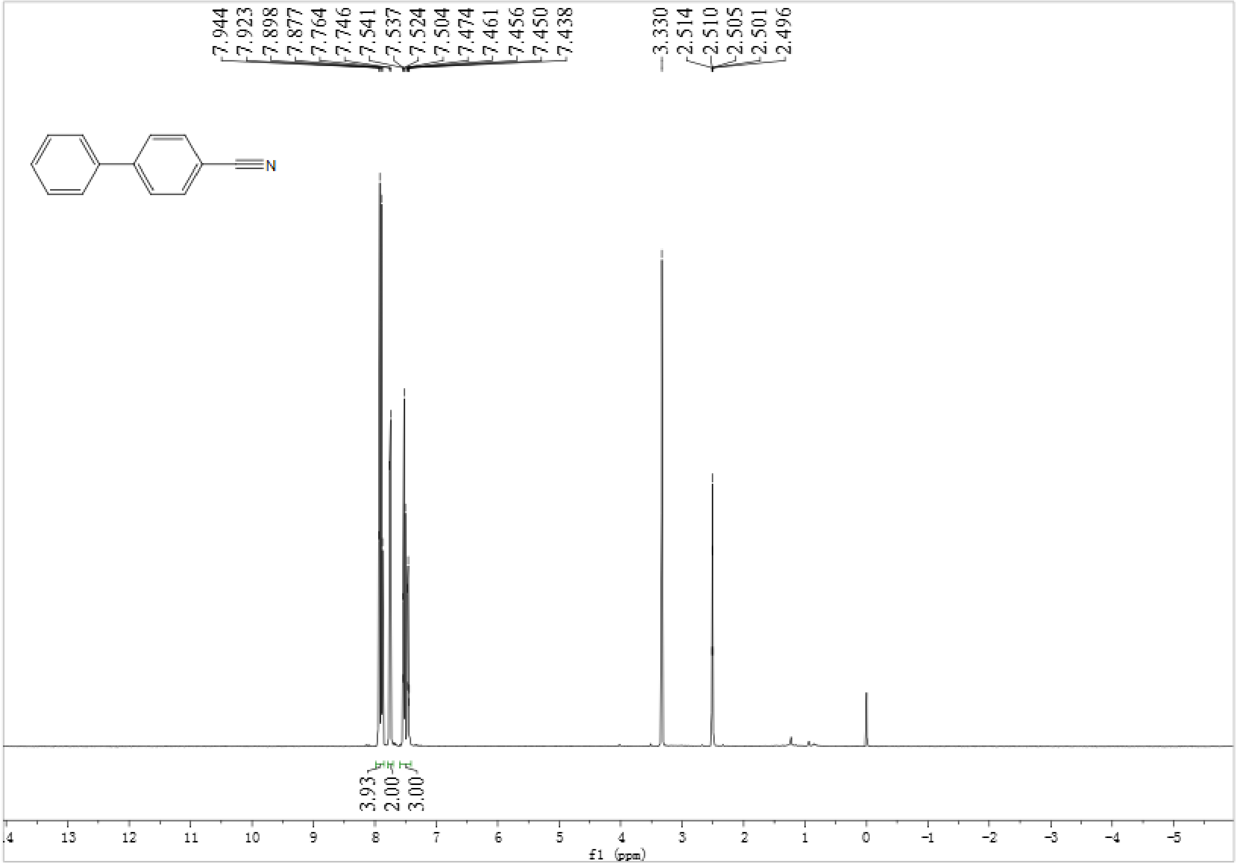

Figure S21. Copy of ¹³C-NMR for 4-cyanobiphenyl.

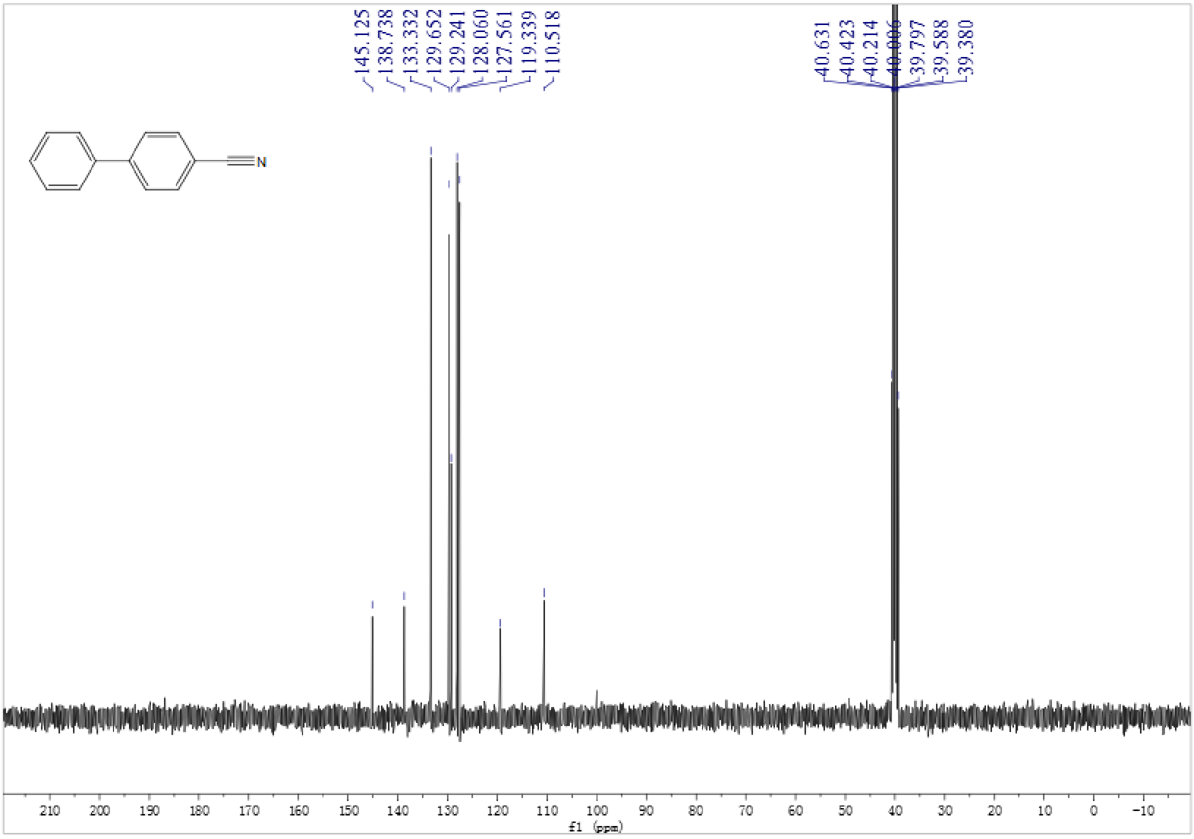

Figure S22. Copy of ¹H-NMR for 4-nitrobiphenyl.

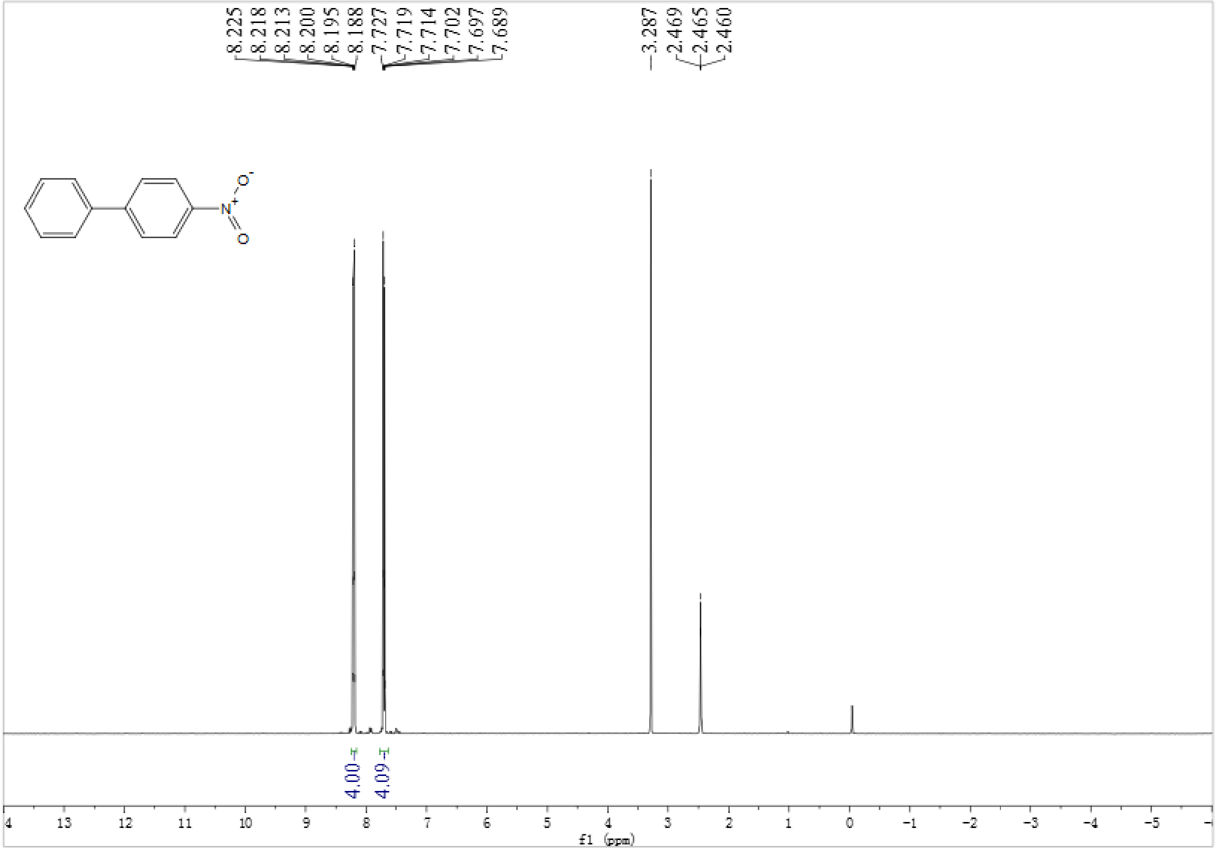

Figure S23. Copy of ¹³C-NMR for 4-nitrobiphenyl.

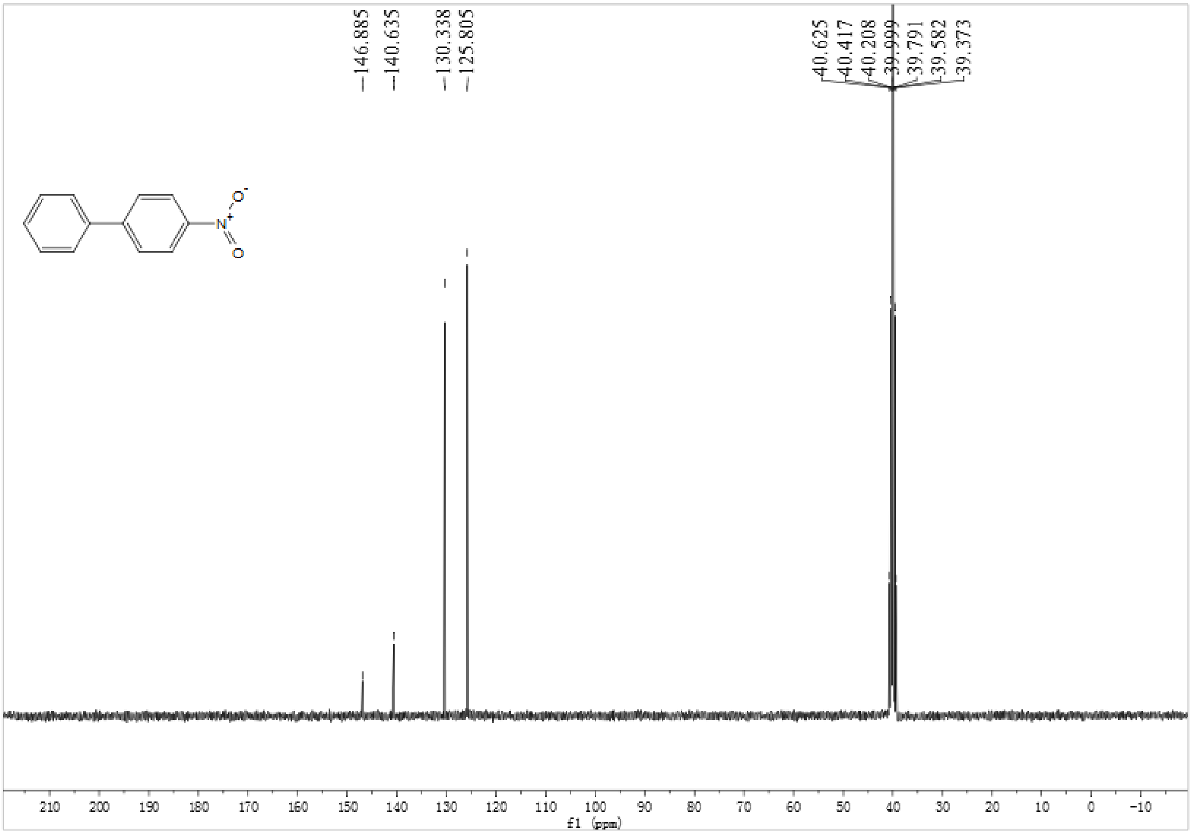

Figure S24. Copy of ^{1}H-NMR for Methyl biphenyl-4-carboxylate.

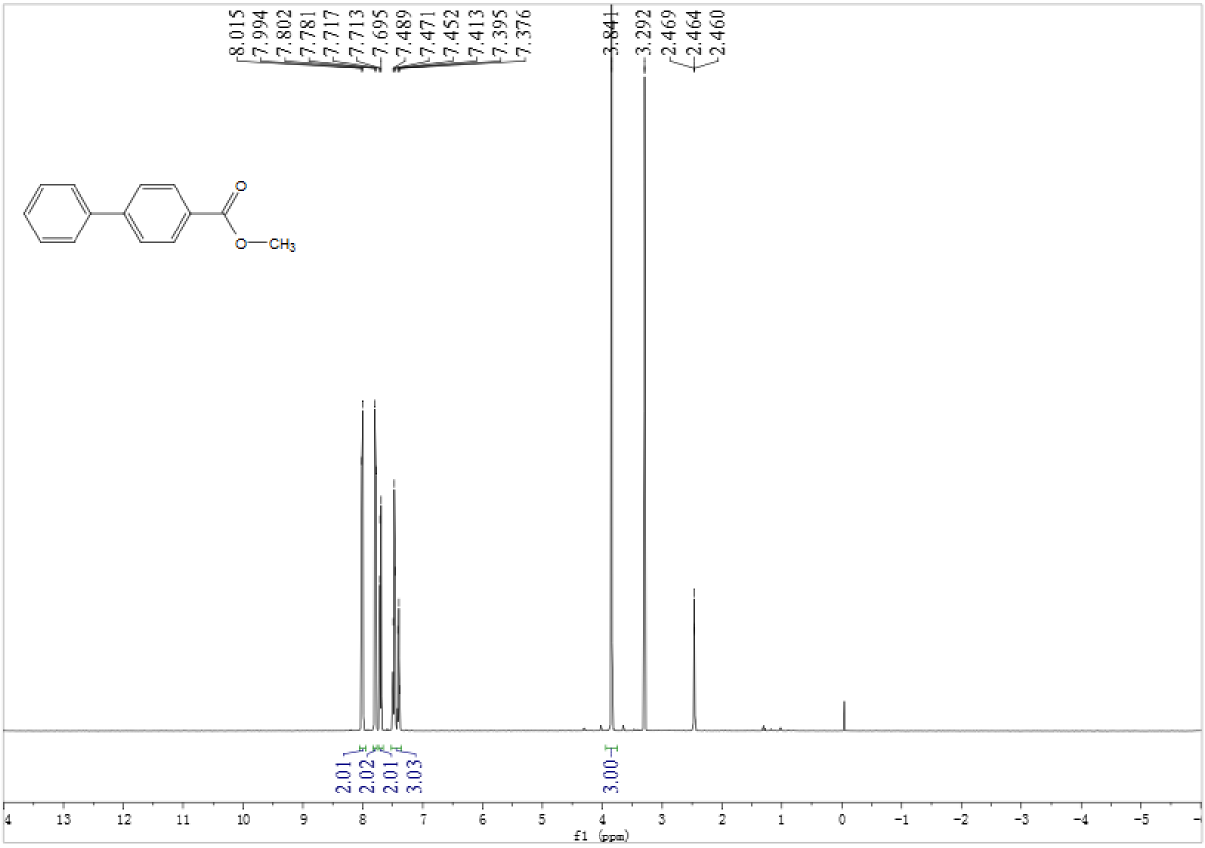

Figure S25. Copy of ^{13}C-NMR for Methyl biphenyl-4-carboxylate.

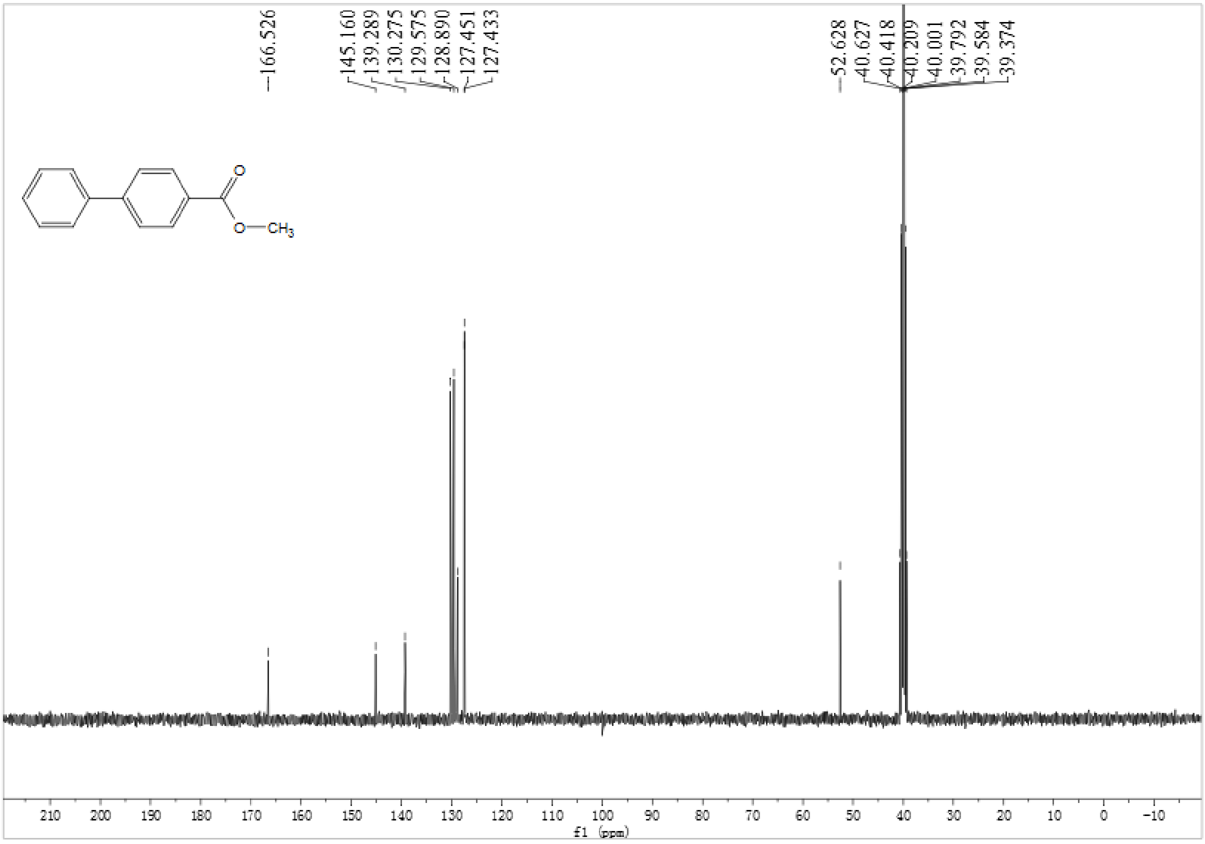

Figure S26. Copy of ^{1}H-NMR for 4-acetylbiphenyl.

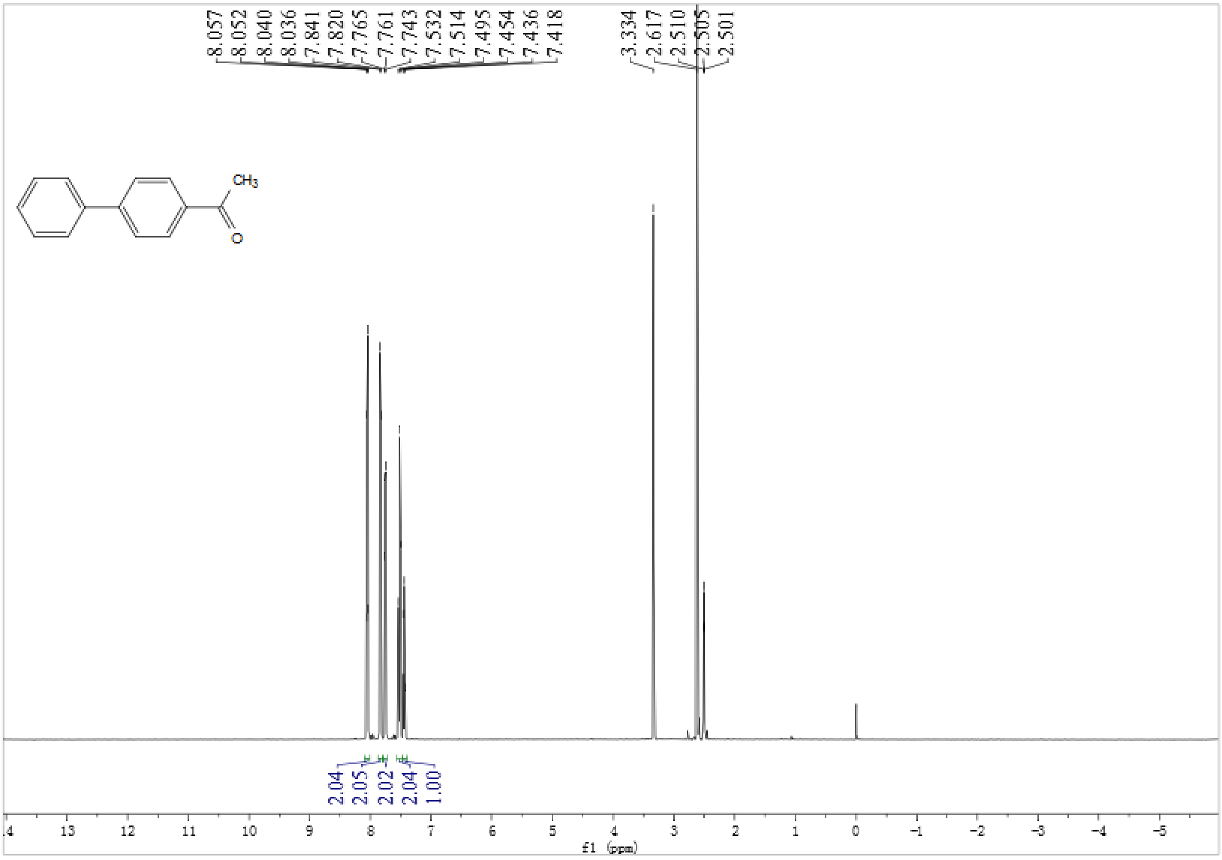

Figure S27. Copy of ^{13}C-NMR for 4-acetylbiphenyl.

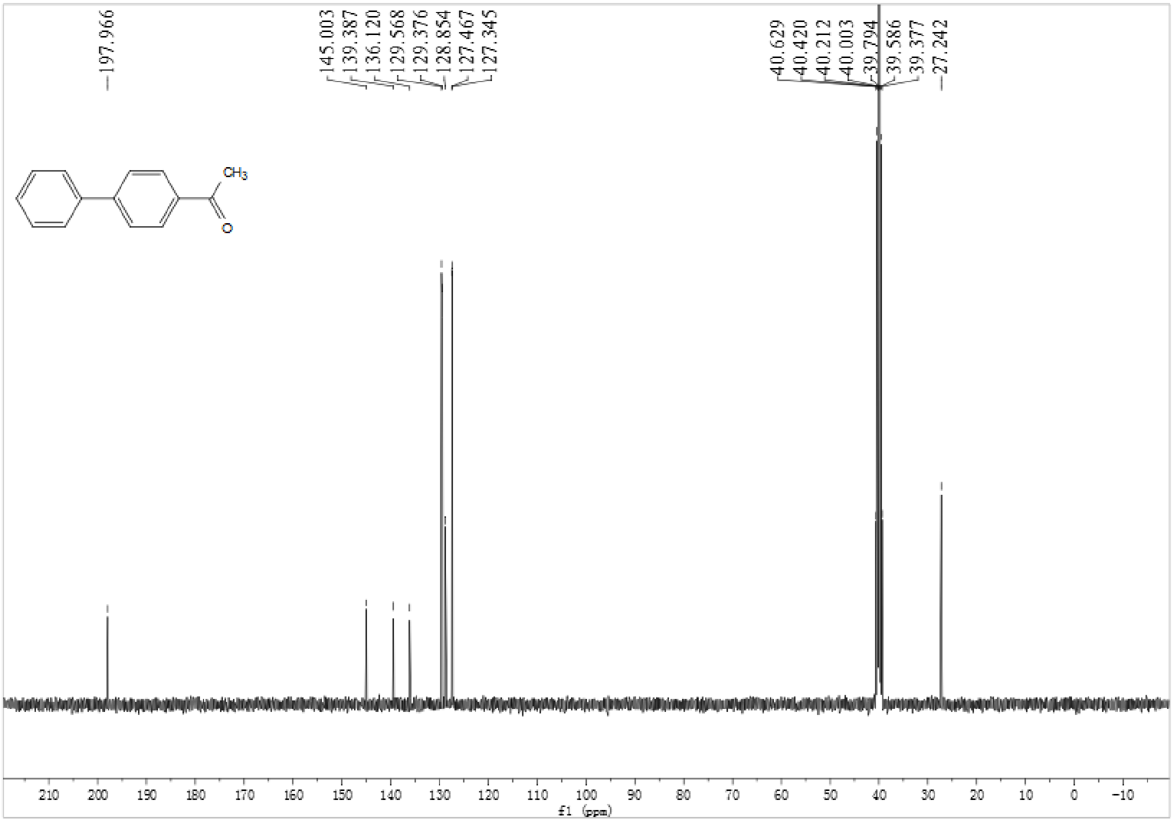

Supplement: Supplementary file 1 [file molecules-19-06524-s001.pdf]
